# Supplementary material for: Identification of New Antifungal Agents Targeting Chitin Synthesis by a Chemical-Genetic Method
Source: Molecules. 2019 Aug 29;24(17):3155. doi: 10.3390/molecules24173155 (PMC6749524; doi:10.3390/molecules24173155)
Supplement: Supplementary file 1 [file molecules-24-03155-s001.pdf]

Table 1. The PCR primers used in this study.

**Primer**	**Oligonucleotide（5′ to 3′）**	**Purpose**
CHSl-F	CCCCATCGAAAGGCCCTT	Used to analyze the transcription of
CHSl-R	CCTGATCTGCATAACCATCGC	*CHS1* gene
CHS2-F	CAGTGAGGAGAGTTTGGAGGA	Used to analyze the transcription of
CHS2-R	GAGTTATGGGCACTATCGCG	*CHS2* gene
CHS3-F	CTCCACGCAGCCCATAAATA	Used to analyze the transcription of
CHS3-R	TTTCCTTCTTTGGCATCCCG	*CHS3* gene
18S rRNA-F	GCCAGCGAGTATAAGCCTTG	Used to analyze the transcription of
18S rRNA-R	AGGCCTCACTAAGCCATTCA	*18S rRNA*
